# Exposure-lag-response associations between weather conditions and ankylosing spondylitis: a time series study

**DOI:** 10.1186/s12891-021-04523-y

**Published:** 2021-07-26

**Authors:** Ling Xin, Jian Liu, Yongjian Zhu, Yanyan Fang

**Affiliations:** 1 The First Affiliated Hospital of Anhui University of Chinese Medicine, 117 Mei Shan Road, Shu Shan District, Hefei, Anhui 230031 People’s Republic of China; 2 School of Management, University of Science and Technology of China, 96 Jin Zhai Road, Bao He District, Hefei, Anhui 230026 People’s Republic of China

**Keywords:** Weather conditions, Ankylosing spondylitis, Relative humidity, Temperature, Distributed lag nonlinear model

## Abstract

**Background:**

Patients with ankylosing spondylitis (AS) have reported that their pain becomes worse when the local weather changes. However, there is limited evidence verifying the short-term associations between meteorological factors and outpatient visits for patients with AS. Therefore, this study evaluates this possible association.

**Methods:**

Meteorological data and data on daily AS outpatient visits to a general hospital in Hefei, China, from 2014 to 2019 were collected and analysed. Distributed lag nonlinear models and Poisson regression models were employed to determine the association between weather conditions and outpatient visits; the results were also stratified by gender and age.

**Results:**

High relative humidity is significantly associated with all patient visits in lag 1 (RR = 1.113, 95% CI 1.021 to 1.213) and lag 7 days (RR = 1.115, 95% CI 1.014 to 1.227). A low relative risk to the nadir is observed in lag 4 days (RR = 0.920, 95% CI 0.862 to 0.983). Male and young patients (< 65 years) are more vulnerable to damp weather, and elderly people (≥ 65 years) are significantly affected by high temperatures in lag 7 days (RR = 3.004, 95% CI 1.201 to 7.510).

**Conclusions:**

Our findings suggest a potential relationship between exposure to weather conditions and increased risk of AS outpatient visits. These results can aid hospitals in preparing for and managing hospital visits by AS patients when the local weather conditions change.

**Supplementary Information:**

The online version contains supplementary material available at 10.1186/s12891-021-04523-y.

## Background

Ankylosing spondylitis (AS) is a chronic autoinflammatory rheumatic disease with high morbidity and disability rates [1]. Features such as immunoinflammatory responses and abnormal bone remodelling are AS’s manifestations [2]. Many AS patients believe that their symptoms of pain become worse during changes in local weather conditions. Previous studies have investigated the relationship between joint pain symptoms in AS and several weather factors, including temperature, humidity, rainfall, and atmospheric pressure [3, 4]. However, most of these studies have been based on the use of questionnaires, which can only represent the feelings of patients and their memories, and results are generally inconclusive and remain controversial.

Some studies suggest that the human body and atmospheric conditions are in a continual state of physical and chemical interaction [5], and weather conditions can have positive or negative effects on human health [6, 7]. Many epidemiological studies have focused on the relationship between weather or climate and human health, and the impacts of the following has been studied: weather conditions [8–10], seasonal variations [11, 12], air pollution [13, 14], and airborne allergens [15, 16]. Evidence suggests that exposure to atmospheric changes and climate elements presents significant risks to more highly vulnerable population groups.

Researchers have recently begun to explore the association between meteorological conditions and the healthcare-seeking behaviour of patients with joint pain. Because most patients with joint pain choose to visit the outpatient unit first, outpatient visits are often studied as an important indicator of healthcare-seeking behaviour. Local weather conditions, such as rainfall, have been found to be closely associated with outpatient visits for joint and back pain in a large patient population [17]. However, the effect of weather on AS outpatient visits has not been adequately considered. This time-series study aimed to assess the associations between short-term weather conditions and AS outpatient visits to a hospital in Hefei, China. Changes in the number of AS outpatient visits associated with temperature and relative humidity from 2014 to 2019 were quantitatively assessed, and the impact of gender and age on these effects was also examined.

## Methods

### Study area and population

This study was reviewed and approved by the institutional committee of the First Affiliated Hospital of Anhui University of Chinese Medicine on research ethics (No. 2020AH-08), and it conforms to the ethical guidelines of the 1975 Declaration of Helsinki. The informed consent was waived by the institutional committee of the First Hospital Affiliated to the Anhui University of Chinese Medicine on research ethics because any variables regarding privacy were not collected. We conducted this time-series study in Hefei, the capital of Anhui Province, China (31°52ʹN, 117°17ʹE), which covers 11,445.1 km^2^ and has a resident population of over 8.189 million (2019). Hefei has a subtropical humid monsoon climate with distinctive seasons.

### Data collection

**Fig. 1:**
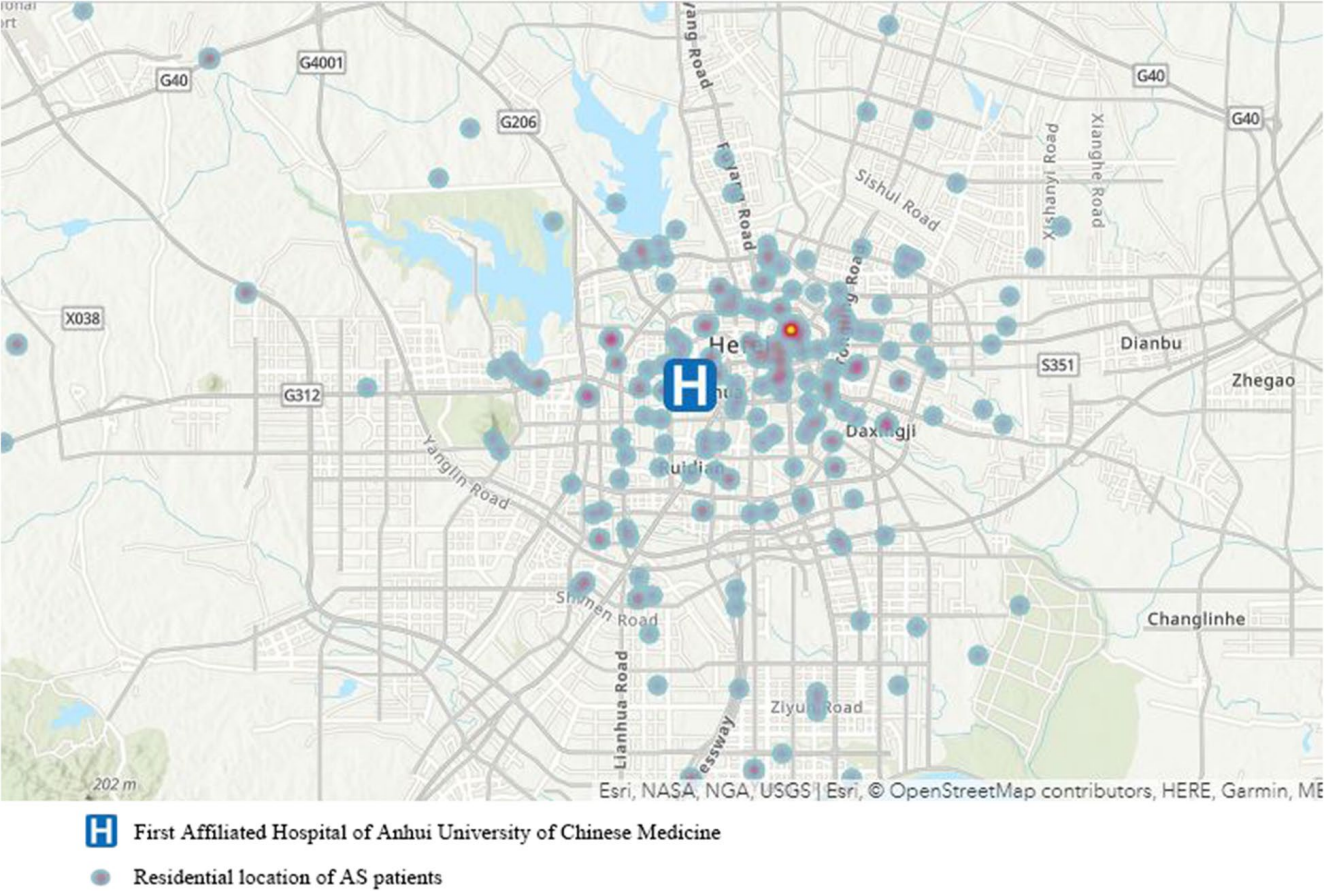
Location of the First Affiliated Hospital of Anhui University of Chinese Medicine and Residential Locations of AS patients

The data of the daily number of outpatients who had been given a primary diagnosis of AS between January 1, 2014, and December 31, 2019, were collected from the First Affiliated Hospital of Anhui University of Chinese Medicine. Patient data included gender, age, residential address, date of visit, diagnosis, and electronic medical record. The diagnostic classification of AS was according to the modified New York criteria [18]. In order to determine the valid outpatient records, we applied the following exclusion criteria: (1) scheduled and regular outpatient visits; (2) follow-up visits; (3) patients who did not take medicine according to the doctor’s advice; (4) patients whose residential addresses were not in Hefei; (5) patients who lacked demographic information (e.g. age, sex); or (6) patients without condition exacerbation. Finally, we included the following outpatient records: (1) patients with condition exacerbation, without therapeutic schedule change; (2) patients with condition exacerbation, and increasing of drug doses or the initiation of a new drug for AS; (3) patients with condition exacerbation, and hospitalisation after visits. The whole data selection process was shown in Supplementary Figures S1. Locations of the hospital and patient residences are given in Fig. 1.

The meteorological data were obtained from the National Meteorological Information Center (http://data.cma.cn), and these included daily average temperature (°C), relative humidity (%), atmospheric pressure (hPa), and rainfall (mm), as the association between these and the exacerbation of AS symptoms has been previously noted by researchers.

### Statistical analysis

AS outpatient visits and daily meteorological data were analysed using stratification factors to obtain descriptive statistics. The correlations between weather conditions during the study period were estimated using Spearman’s correlation coefficients because all these variables were not normally distributed. The daily number of AS outpatient visits was linked with daily meteorological factors by date and then analysed for exposure–response associations. As the daily count of outpatients approximately followed a Poisson distribution, we estimated the short-term association between meteorological factors and outpatient visits by conducting a quasi-Poisson regression analysis using a distributed lag nonlinear model (DLNM) [19],

$$\mathrm{log}\left[E\left({Y}_{t}\right)\right]=\alpha +\beta {TEM}_{t,l}+ns\left({Time}_{t}, 8\times 6\right)+ns\left({RHU}_{t}, 3\right)+ns\left({PRS}_{t}, 3\right)+ns\left({RAF}_{t}, 3\right)+{\gamma DOW}_{t}+{\delta Holiday}_{t},$$

where ${Y}_{t}$ represents the observed count of AS outpatient visits at day t; $\mathrm{\alpha }$ is the intercept, $\mathrm{l}$ is the lag days, ${TEM}_{t,l}$ is the cross-basis function for mean temperature, and $\mathrm{ns}\left({Time}_{t}, 8\times 6\right)$ represents a natural cubic spline with 8 degrees of freedom (df) per year for the time taken to control for long-term and seasonality trends. Other environmental confounding variables are adjusted for using a natural cubic spline with 3 df for relative humidity (${RHU}_{\mathrm{t}}$), atmospheric pressure (${PRS}_{t}$), and rainfall (${RAF}_{t}$) [20, 21]; the day of the week (${\mathrm{DOW}}_{\mathrm{t}}$: Monday to Sunday) and public holidays (${\mathrm{Holiday}}_{\mathrm{t}}$: yes, no) are also adjusted as categorical variables. $\beta$, $\gamma$, and $\delta$ are the coefficients for the corresponding variables. The same approach was employed to examine the association between AS outpatient visits and relative humidity, and the model formula was defined as:

$$\mathrm{log}\left[E\left({Y}_{t}\right)\right]=\alpha +\beta {RHU}_{t,l}+ns\left({Time}_{t}, 8\times 6\right)+ns\left({TEM}_{t}, 3\right)+ns\left({PRS}_{t}, 3\right)+ns\left({RAF}_{t}, 3\right)+{\gamma DOW}_{t}+{\delta Holiday}_{t}.$$

We modelled the exposure–response relationship, taking each variable’s mean values as the reference value [22]. The effects of low and high weather variables were estimated by calculating the risk of AS outpatient visits at the 1st and 99th percentiles of variables relative to the mean values [21].

It was reported that rheumatic patients would exacerbate symptoms within one week after temperature and humidity change, and we assessed the lagged effect up to 7 days following the start of these two variables to explore whether the effect of the weather condition was immediate or delayed [23, 24]. Furthermore, we explored the potential effect of AS risk modification using age (< 65 years and ≥ 65 years) and gender (male and female) [24].

We conducted various sensitivity analyses, varying the df (7–9 per year) for time to control for long-term and seasonality trends as well as df (3–5) for potential confounding environmental factors to test the robustness of our results. *P* < 0.05 was considered statistically significant in all analyses. All statistical analyses were conducted with R software (version 3.6.3) using the package dlnm (version 2.4.2).

## Results

**Fig. 2:**
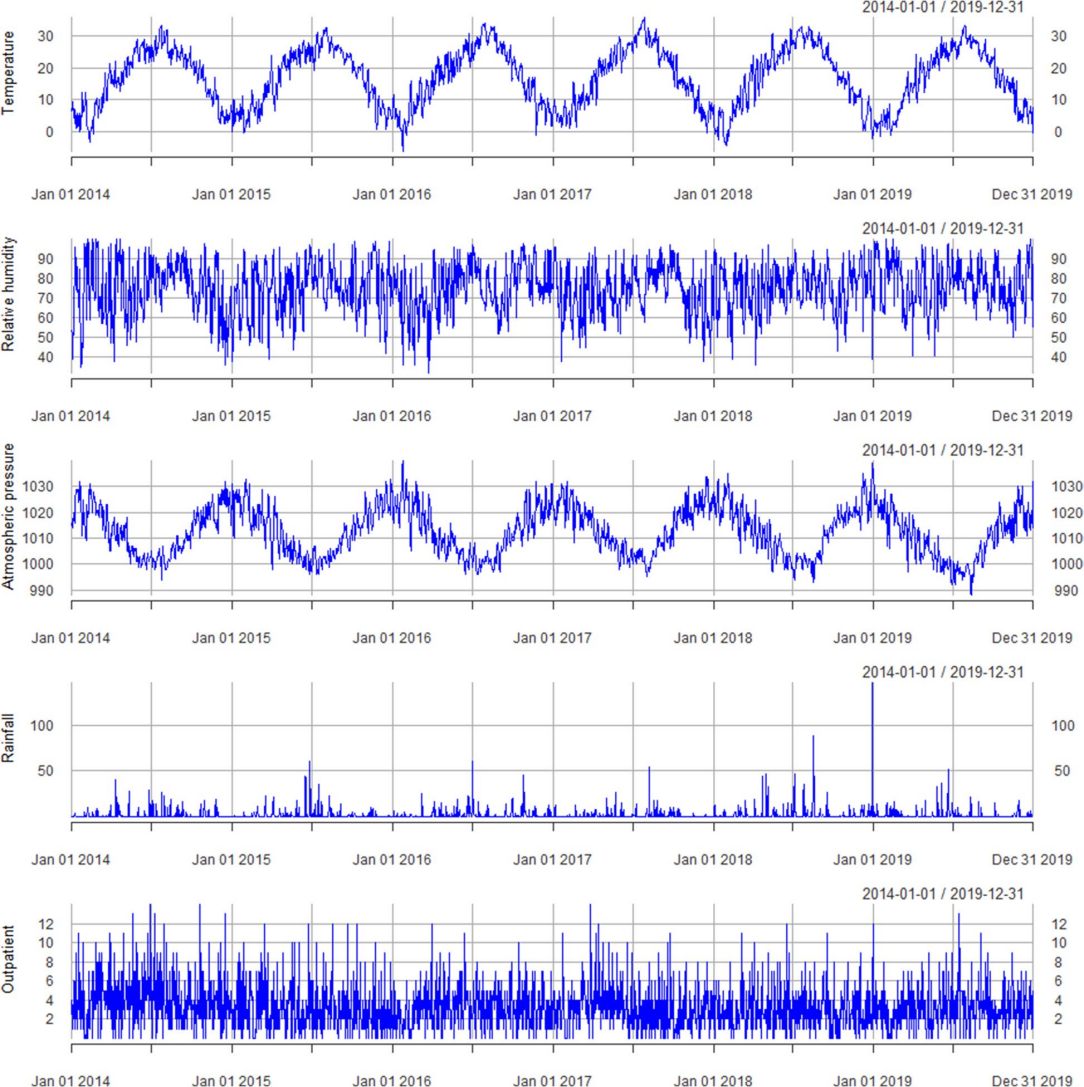
Time-series distribution of daily temperature, relative humidity, wind speed, atmospheric pressure, rainfall, and AS outpatient visits in Hefei, China, from January 2014 to December 2019

Tables 1 and 2 present a summary of the statistics associated with the demographics of AS outpatient visit data and meteorological data obtained during the study period in Hefei, China. Patients were found to be predominantly male (70.57%) and younger than 65 years old (96.83%). Average daily temperature, relative humidity, and atmospheric pressure were 16.90 °C, 75.02%, 1012.51 hPa, respectively. The median rainfall was 0.00 mm. The time-series distribution of these weather variables and outpatient visits are shown in Fig. 2.

**Table 1:** Demographic characteristics of AS patient visiting the First Affiliated Hospital of Anhui University of Chinese Medicine in Hefei, China, from January 2014 to December 2019

	Number of cases	Percentage (%)
Outpatient	7058	
Age, years (Mean ± SD)	37.44 ± 12.06	
0–64 years	6834	96.83
65 + years	224	3.17
Gender		
Male	4981	70.57
Female	2077	29.43
Season		
Spring (Mar–May)	1973	27.95
Summer (Jun–Aug)	1882	26.66
Autumn (Sep–Nov)	1643	23.28
Winter (Dec–Feb)	1560	22.11

**Table 2:** Statistics of meteorological variables in Hefei, China, from January 2014 to December 2019

	Mean	SD	Min	P25	P50	P75	Max
Temperature (°C)	16.90	9.17	–6.00	8.80	17.80	24.55	35.60
Relative humidity (%)	75.02	12.73	32.00	67.00	76.00	85.00	100
Atmospheric pressure (hPa)	1012.51	9.53	988.00	1004.00	1012.00	1020.00	1040.00
Rainfall (mm)	–	–	0.00	0.00	0.00	0.50	146.00

The ‘rainfall’ was not disturbed normally

Table 3 shows the correlation coefficients of several of the meteorological variables. Average temperature was positively correlated with relative humidity (*r* = 0.07, *P* = 0.001) and negatively correlated with atmospheric pressure (*r* = -0.90, *P* < 0.001). Relative humidity was negatively correlated with atmospheric pressure (*r* = -0.22, *P* < 0.001), but positively correlated with rainfall (*r* = 0.64, *P* < 0.001). Atmospheric pressure and rainfall were negatively correlated with each other (*r* = -0.11, *P* < 0.001).

**Table 3:** Spearman correlation coefficients between meteorological variables in Hefei, China, from January 2014 to December 2019

Variables		*r*	*P*
TEM	RHU	0.07	0.001
TEM	PRS	–0.90	< 0.001
TEM	RAF	–0.04	0.071
RHU	PRS	–0.22	< 0.001
RHU	RAF	0.64	< 0.001
PRS	RAF	–0.11	< 0.001

*TEM* Temperature, *RHU* Relative humidity, *PRS* Atmospheric pressure, *RAF* Rainfall

**Fig. 3:**
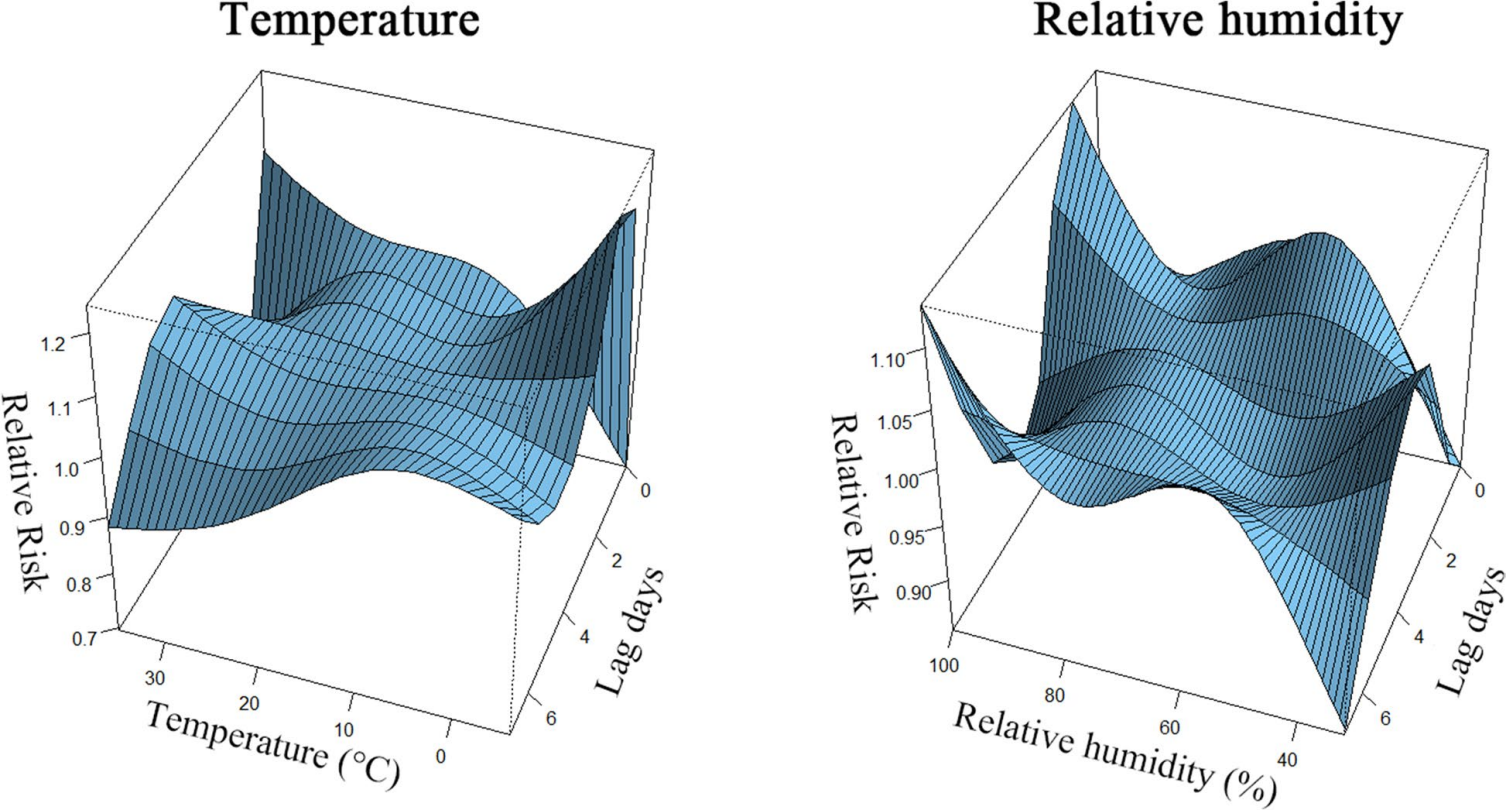
Bi-dimensional exposure-lag-response surfaces between each meteorological variable and AS outpatient visits

The bi-dimensional exposure-lag-response associations between weather variables and outpatient visits for AS are shown in Fig. 3. The results show nonlinear relationships between weather variables’ values and outpatient visits.

**Fig. 4:**
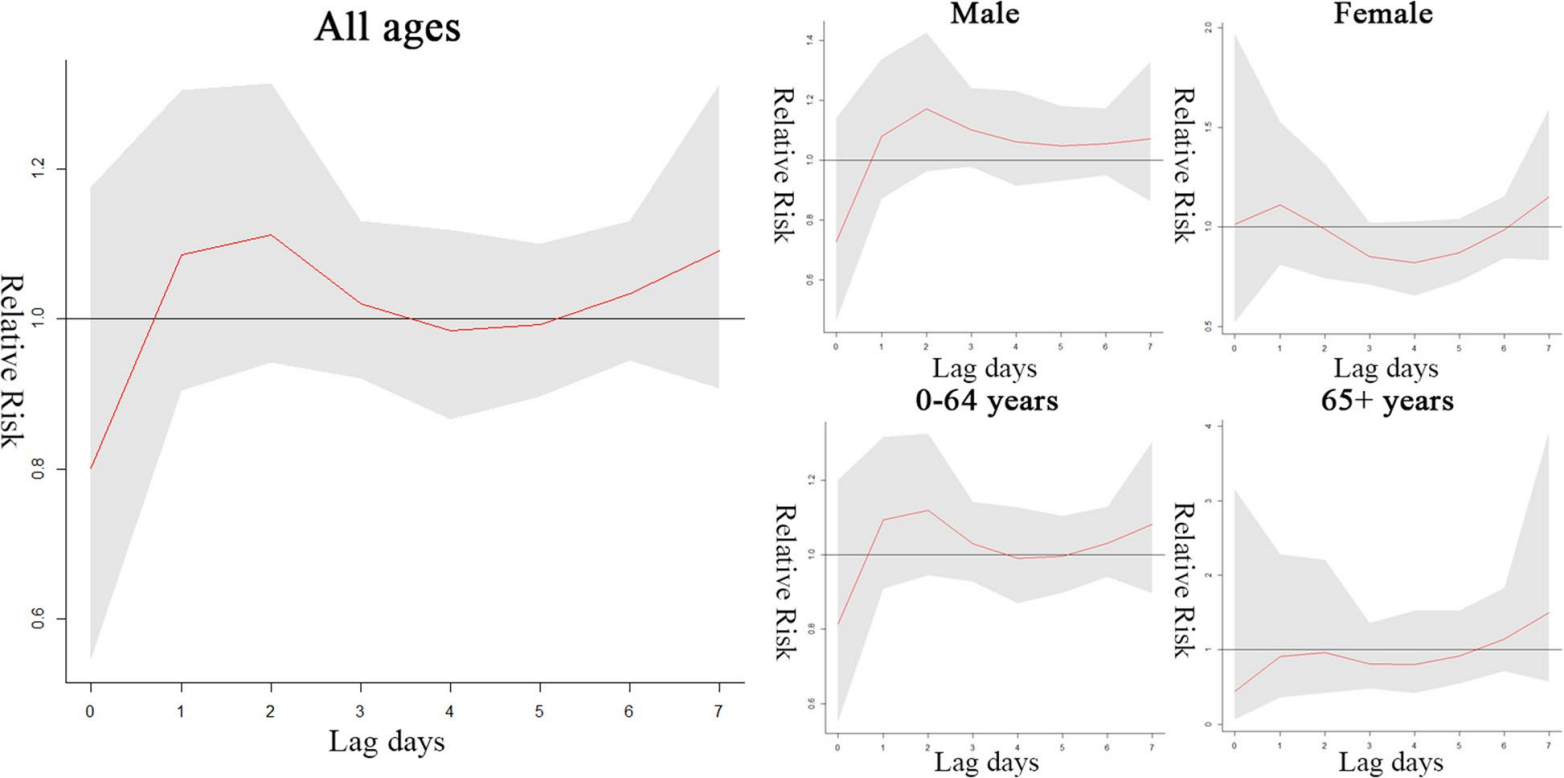
Effects of low temperature (-1 °C) on AS outpatient visits on lag days 0–7

**Fig. 5:**
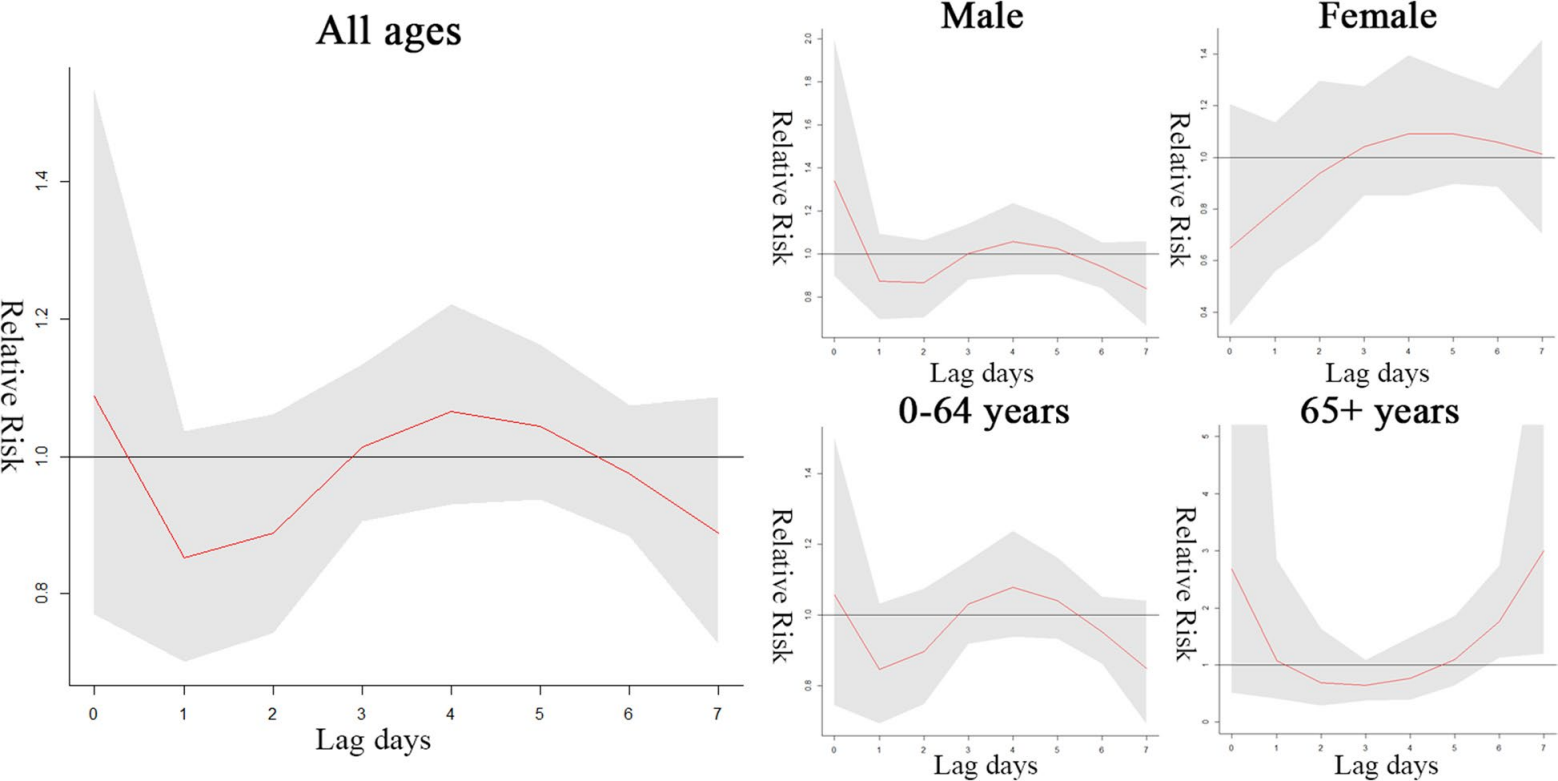
Effects of high temperature (32.9 °C) on AS outpatient visits on lag days 0–7

Figures 4 and 5 show quantitative estimates of the effects of low and high temperature on outpatient visits for AS in different subgroups and on different lag days. The specific values are shown in Supplementary Tables S1. It is considered likely that high temperature in the previous one week affects the healthcare-seeking behaviour of elderly patients (lag 6: RR = 1.761, 95% CI 1.130 to 2.744; lag 7: RR = 3.004, 95% CI 1.201 to 7.510), but no statistically significant relationship is evident between low temperature and AS cases in any of the population groups. This indicated that, relative to 16.90 °C, high temperature (32.9 °C) was associated with a 76.1% increase in the number of elderly outpatient visits on lag 6 days. The correlation between temperature and atmospheric pressure was high, thus the RR value was analysed without atmospheric pressure, and no significant difference was found.

**Fig. 6:**
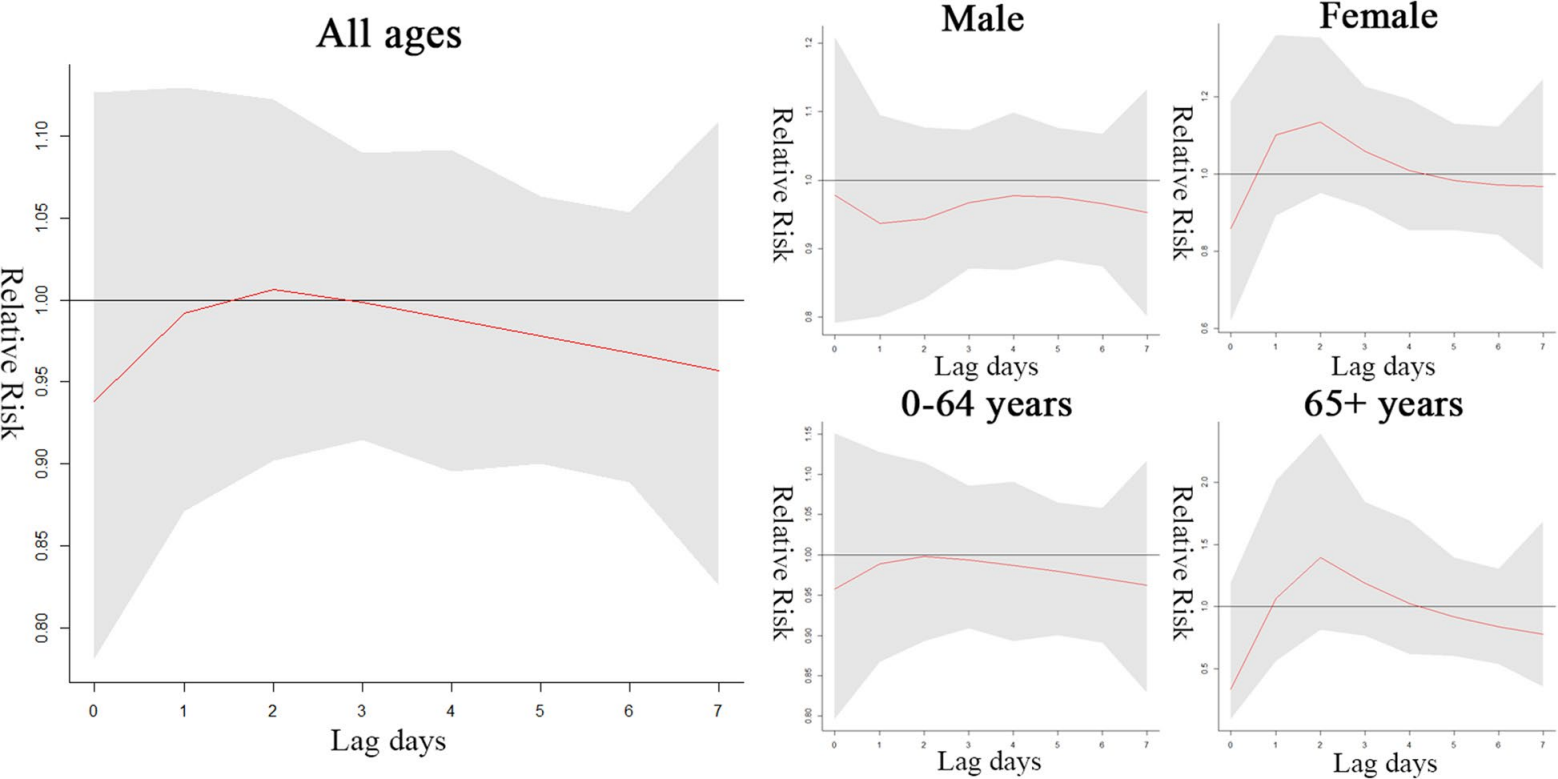
Effects of low relative humidity (42%) on AS outpatient visits on lag days 0–7

**Fig. 7:**
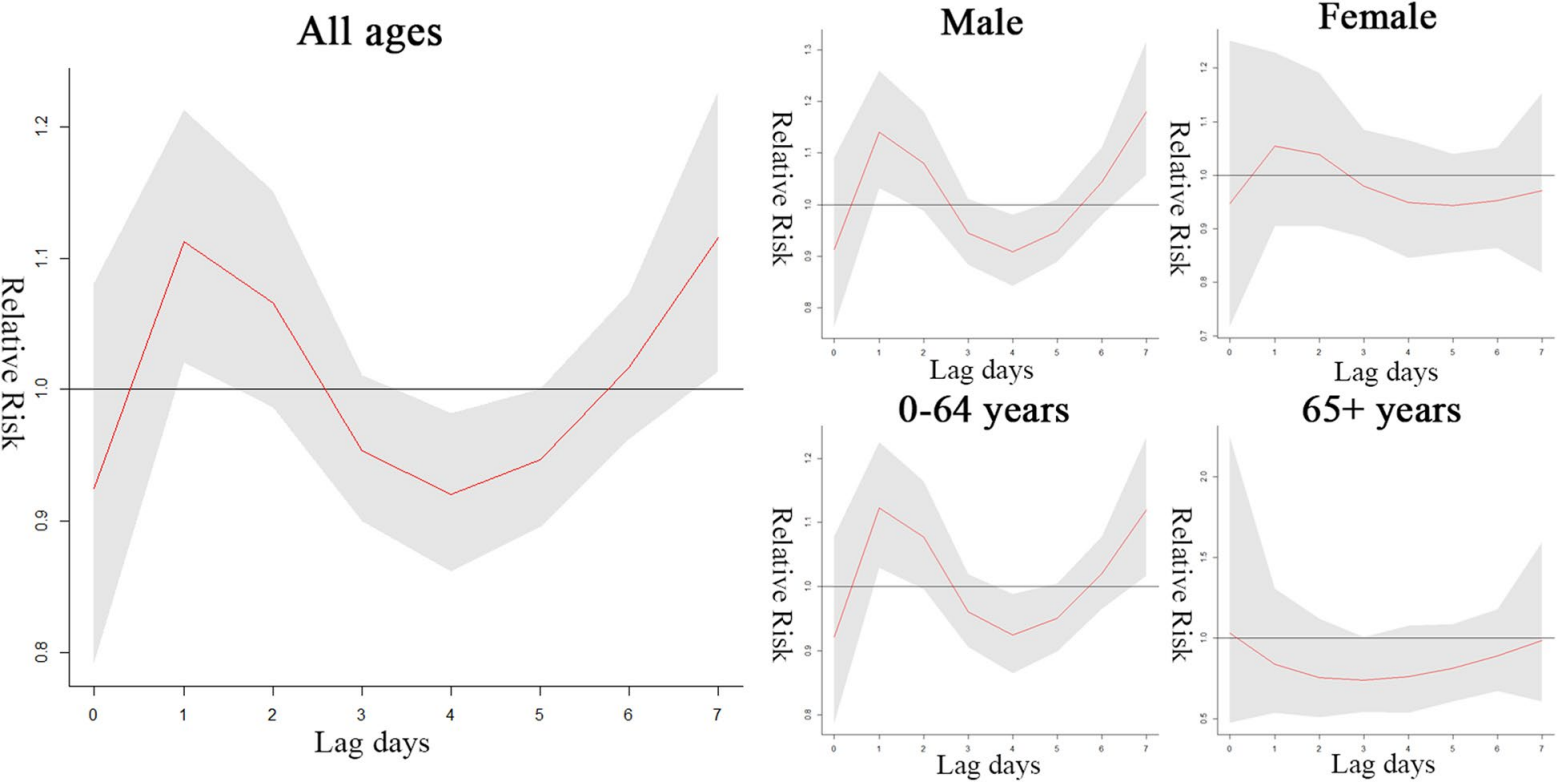
Effects of high relative humidity (98%) on AS outpatient visits on lag days 0–7

There is no statistically significant association between low relative humidity and outpatient visits (Fig. 6). Figure 7 shows that high relative humidity was significantly associated with all patient visits on lag 1 (RR = 1.121, 95% CI 1.029 to 1.221) and lag 7 days (RR = 1.121, 95% CI 1.020 to 1.232), predominantly for male patients and young patients, and high relative humidity showed a negative association with the nadir on lag 4 days (RR = 0.916, 95% CI 0.858 to 0.978). The specific values are shown in Supplementary Table S2.

The sensitivity analysis results are shown in the supplementary material (Supplementary Figures S2–S17). Generally, the effects of environmental factors on outpatient visits for AS were robust to variations in the df for time trends and meteorological factors.

## Discussion

A time-series study was conducted to investigate the association between 7,058 AS outpatient visits and certain meteorological factors during 2014 and 2019 in Hefei city, China. A relationship was found between a high humidity event and an increase in the number of AS patients visiting the hospital the following day or seven days later. Males and young patients are more susceptible to a rise in humidity than other groups. A significant positive association was also observed between high temperature events and elderly patients attending hospital on lag 6 and 7 days.

To the best of our knowledge, few studies have analysed the relationship between AS and meteorological factors; although, research has investigated the relationship between weather conditions and rheumatic pain. For example, a questionnaire survey among 394 rheumatic patients in Romania suggested that most people reported an intensification in pain when the weather deteriorated, and this was significant with an increase in humidity or decrease in temperature [25]. A retrospective cohort study concluded that rainfall exacerbated the joint pain of patients with rheumatoid arthritis to the extent that some were re-admitted to the hospital [24].

However, the questionnaire surveys and hospital admission numbers enable only a small proportion of populations affected by weather conditions to be detected because only people with severe disease or specific conditions tend to be included [26]. According to the 2019 China Health Statistics Yearbook, hospital outpatient and emergency visits accounted for nearly 95% of the total number of hospital visits, while hospital admissions only accounted for 2.4%. It is also notable that the number of patients hospitalised may not vary appreciably from day to day because people in China prefer to be admitted to high-level hospitals, so approximating a saturation for inpatient beds [27]. Therefore, we investigated outpatient and emergency visits instead of hospital admissions, which yield high levels of coverage by including cases with less severe diseases or conditions [26].

The results of this study suggest that high humidity leads to a lagged increase in outpatient visits. Previous studies have also shown that rainy or damp weather can exacerbate joint pain symptoms, acting as a trigger for patients compelling them to attend the hospital [17, 24]. One theory suggests that tendons, muscles, bones, and areas of scarring have different densities and that cold and damp weather has different effects on the expansion and contraction of different tissue types, which can cause micro-trauma and pain [28]. Another related study indicated that ambient temperature and high relative humidity can increase the expression of VEGF and IL-1 in articular cartilage, which might influence the promotion, pathological course, and severity of AS in patients [29]. We found a significant increase in the number of patients attending the hospital on the second and seventh days after the onset of high humidity. However, 4 days after the onset, the number of AS outpatients reached a nadir point. The reasons for such intriguing results were further assessed.

Xie et al. [24] believe that rainy or damp weather not only worsens the symptoms of joint pain but also makes it more difficult for patients to visit the hospital. Therefore, patients that are severely affected wait to see a doctor on the following day, and patients with less pain choose to make an appointment for outpatient service one week later. It has also been reported that significantly more people attend emergency departments on fair weather days (warm, dry, and sunny) than on bad weather days (cool, rainy, and dull) [30], which is consistent with our interpretation. However, it remains unclear why outpatient visits decreased on the fourth day following the event. We speculate that the hospital patient population is relatively fixed and that most patients may not re-visit a doctor in the short term after treatment. As a result, the number of AS outpatient visits is expected to decrease significantly after the peak. However, this remains inconclusive and requires further investigation considering wider populations. The gender and age stratifying analysis showed that the behaviour of only male and young patients (< 65 years) was consistent with this result, as they may be more vulnerable to damp environments because they are more likely to be outdoors [31].

In contrast with previous studies, we found no significant relationship between low temperature and AS outpatient visits. However, 6 to 7 days after a high temperature event, the number of elderly patients increased sharply. Most people in China have air conditioning; therefore, low temperatures do not have a significant impact on people. However, air conditioning can cause the environment to become cold in summer, which could exacerbate symptoms in elderly patients. Fernandes et al. [32] provided evidence that such a change in temperature could increase blood flow and affect the sensitivity of arthritis patients to pain. In this respect, elderly people over the age of 65 are more vulnerable and sensitive [33].

Our study has several limitations. As in most previous time-series studies, we averaged the measurements across various fixed-site stations as the proxy for the population exposure level to weather conditions in Hefei. The simple averaging method could result in apparent exposure measurement errors. Additionally, monitoring measurements can differ from location to location and on a personal exposure level [27]. Another limitation is that we lacked the clinical data associated with disease activity and could only use the exclusion criteria and identified outpatient record categories to determine the valid outpatient records. Finally, we conducted our study in one hospital within a city, which reduces the generalisability of our results in other contexts; therefore, caution should be practiced when generalising our results for other areas or populations.

## Conclusion

AS is a painful disease, and associated medical costs are high. We herein investigated the evidence for a significant and nonlinear relationship between weather conditions and outpatient visits for AS using a time-series method. This study suggests that damp weather may cause an increase in the number of AS patients visiting the hospital. However, the effects differ depending on age and gender. The results show that 6 to 7 days after a high temperature event, there may be a surge in the number of elderly patients visiting hospitals. We suggest that policymakers reformulate their policies on exposure to weather conditions to assist hospitals in managing AS patient visits, and physicians could consider environmental factors in treatments for AS patients.

## Supplementary Information

**Additional file 1: Fig. S1.** Data selection process. **Fig. S2.** Effects for cold weather (1st percentiles of temperature) on daily outpatient visits for AS at lag 0–7 day; A nature cubic spline with 3 dfs was used to control environmental factors, 7 dfs per year was used to control the seasonal patterns and long term trends. **Fig. S3.** Effects for cold weather (1st percentiles of temperature) on daily outpatient visits for AS at lag 0–7 day; A nature cubic spline with 3 dfs was used to control environmental factors, 9 dfs per year was used to control the seasonal patterns and long term trends. **Fig. S4.** Effects for cold weather (1st percentiles of temperature) on daily outpatient visits for AS at lag 0–7 day; A nature cubic spline with 4 dfs was used to control environmental factors, 8 dfs per year was used to control the seasonal patterns and long term trends. **Fig. S5.** Effects for cold weather (1st percentiles of temperature) on daily outpatient visits for AS at lag 0–7 day; A nature cubic spline with 5 dfs was used to control environmental factors, 8 dfs per year was used to control the seasonal patterns and long term trends. **Fig. S6.** Effects for hot weather (99st percentiles of temperature) on daily outpatient visits for AS at lag 0–7 day; A nature cubic spline with 3 dfs was used to control environmental factors, 7 dfs per year was used to control the seasonal patterns and long term trends. **Fig. S7.** Effects for hot weather (99st percentiles of temperature) on daily outpatient visits for AS at lag 0–7 day; A nature cubic spline with 3 dfs was used to control environmental factors, 9 dfs per year was used to control the seasonal patterns and long term trends. **Fig. S8.** Effects for hot weather (99st percentiles of temperature) on daily outpatient visits for AS at lag 0–7 day; A nature cubic spline with 4 dfs was used to control environmental factors, 8 dfs per year was used to control the seasonal patterns and long term trends. **Fig. S9.** Effects for hot weather (99st percentiles of temperature) on daily outpatient visits for AS at lag 0–7 day; A nature cubic spline with 5 dfs was used to control environmental factors, 8 dfs per year was used to control the seasonal patterns and long term trends. **Fig. S10.** Effects for dry weather (1st percentiles of relative humidity) on daily outpatient visits for AS at lag 0–7 day; A nature cubic spline with 3 dfs was used to control environmental factors, 7 dfs per year was used to control the seasonal patterns and long term trends. **Fig. S11.** Effects for dry weather (1st percentiles of relative humidity) on daily outpatient visits for AS at lag 0–7 day; A nature cubic spline with 3 dfs was used to control environmental factors, 9 dfs per year was used to control the seasonal patterns and long term trends. **Fig. S12.** Effects for dry weather (1st percentiles of relative humidity) on daily outpatient visits for AS at lag 0–7 day; A nature cubic spline with 4 dfs was used to control environmental factors, 8 dfs per year was used to control the seasonal patterns and long term trends. **Fig. S13.** Effects for dry weather (1st percentiles of relative humidity) on daily outpatient visits for AS at lag 0–7 day; A nature cubic spline with 5 dfs was used to control environmental factors, 8 dfs per year was used to control the seasonal patterns and long term trends. **Fig. S14.** Effects for damp weather (99th percentiles of relative humidity) on daily outpatient visits for AS at lag 0–7 day; A nature cubic spline with 3 dfs was used to control environmental factors, 7 dfs per year was used to control the seasonal patterns and long term trends. **Fig. S15.** Effects for damp weather (99th percentiles of relative humidity) on daily outpatient visits for AS at lag 0–7 day; A nature cubic spline with 3 dfs was used to control environmental factors, 9 dfs per year was used to control the seasonal patterns and long term trends. **Fig. S16.** Effects for damp weather (99th percentiles of relative humidity) on daily outpatient visits for AS at lag 0–7 day; A nature cubic spline with 4 dfs was used to control environmental factors, 8 dfs per year was used to control the seasonal patterns and long term trends. **Fig. S17.** Effects for damp weather (99th percentiles of relative humidity) on daily outpatient visits for AS at lag 0–7 day; A nature cubic spline with 5 dfs was used to control environmental factors, 8 dfs per year was used to control the seasonal patterns and long term trends.

**Additional file 2: Table S1.** Relative risk (RR) and 95% confidence intervals (95% CI) for low and high temperatures associated with daily AS outpatient over different lag days in Hefei, China, from January 2014 to December 2019. **Table S2.** Relative risk (RR) and 95% confidence intervals (95% CI) for low and high relative humidity associated with daily AS outpatient visits over different lag days in Hefei, China, from January 2014 to December 2019.

## Data Availability

The datasets used and/or analysed during the current study are available from the corresponding author upon reasonable request.

## Abbreviations

AS: Ankylosing spondylitis
RR: Relative risk
CI: Confidence interval
DLNM: Distributed lag nonlinear model
VEGF: Vascular endothelial growth factor
IL-1: Interleukin-1
