# Supplementary material for: Exposure-lag-response associations between weather conditions and ankylosing spondylitis: a time series study
Source: BMC Musculoskelet Disord. 2021 Jul 26;22:641. doi: 10.1186/s12891-021-04523-y (PMC8314534; doi:10.1186/s12891-021-04523-y)

**Fig. S1** Data selection process


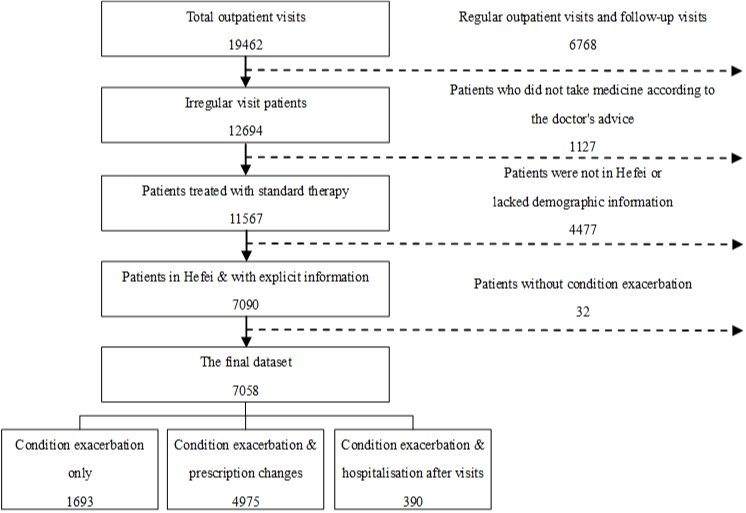


**Fig. S2** Effects for cold weather (1st percentiles of temperature) on daily outpatient visits for AS at lag 0-7 day; A nature cubic spline with 3 dfs was used to control environmental factors, 7 dfs per year was used to control the seasonal patterns and long term trends.


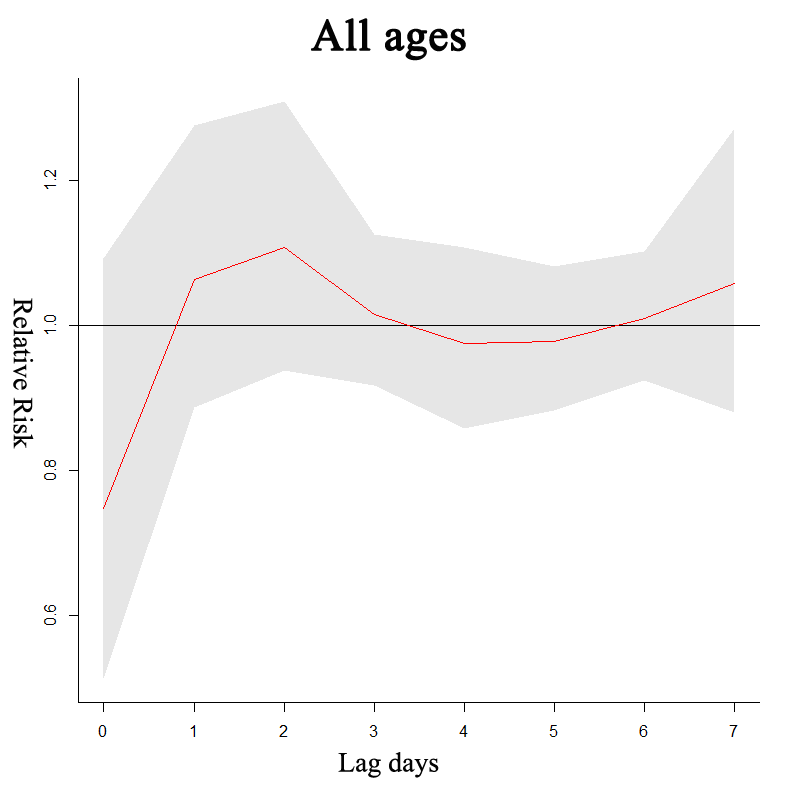


**Fig. S3** Effects for cold weather (1st percentiles of temperature) on daily outpatient visits for AS at lag 0-7 day; A nature cubic spline with 3 dfs was used to control environmental factors, 9 dfs per year was used to control the seasonal patterns and long term trends.


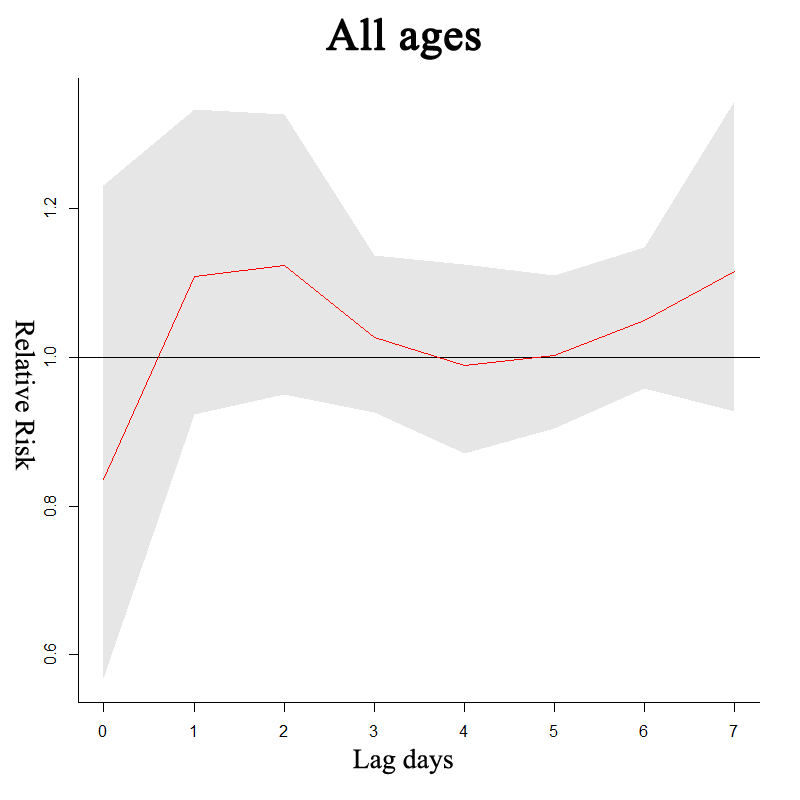


**Fig. S4** Effects for cold weather (1st percentiles of temperature) on daily outpatient visits for AS at lag 0-7 day; A nature cubic spline with 4 dfs was used to control environmental factors, 8 dfs per year was used to control the seasonal patterns and long term trends.


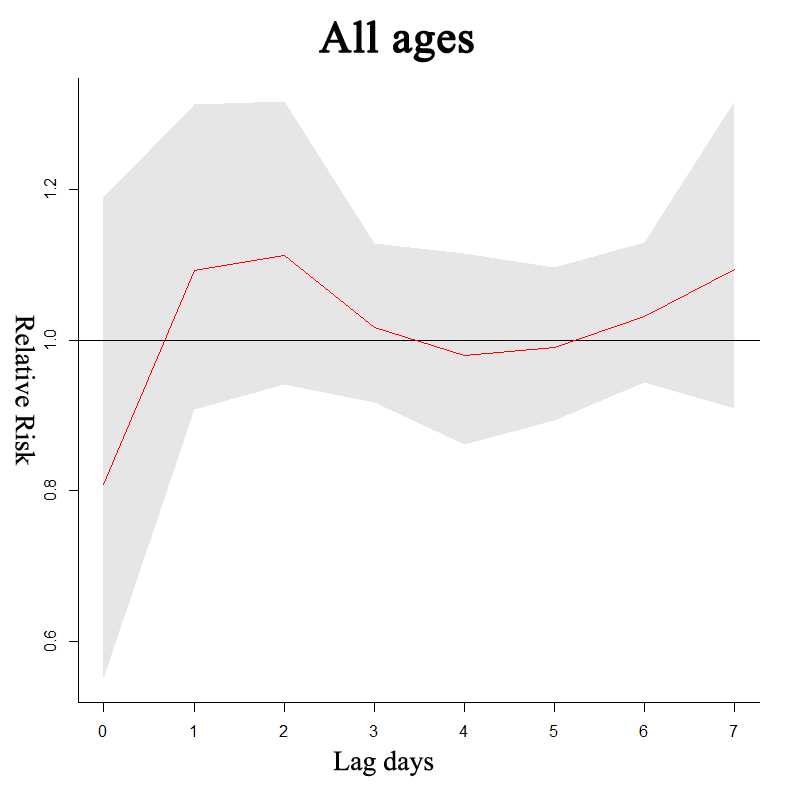


**Fig. S5** Effects for cold weather (1st percentiles of temperature) on daily outpatient visits for AS at lag 0-7 day; A nature cubic spline with 5 dfs was used to control environmental factors, 8 dfs per year was used to control the seasonal patterns and long term trends.


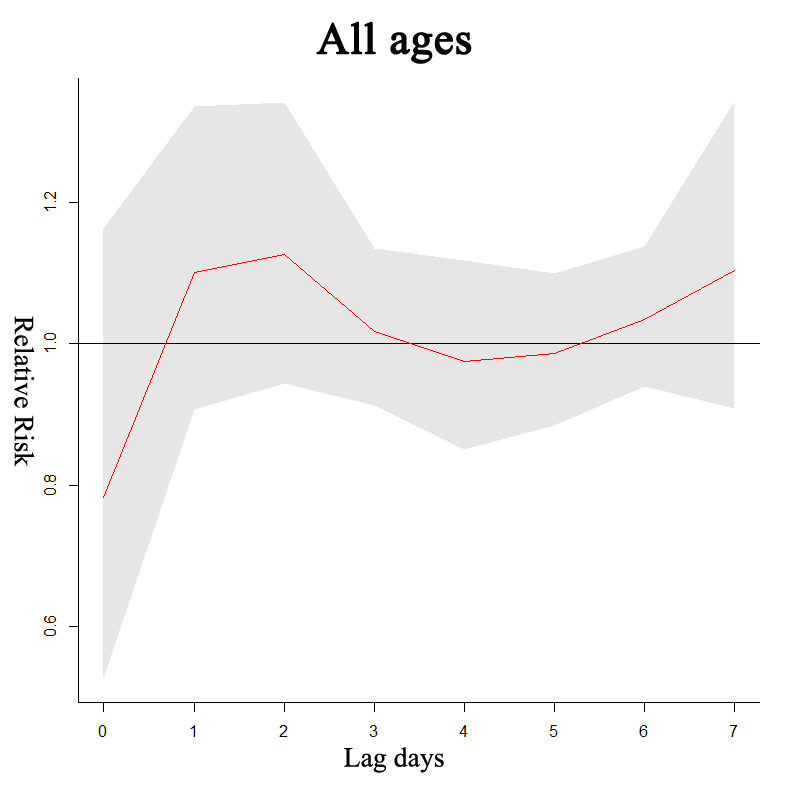


**Fig. S6** Effects for hot weather (99st percentiles of temperature) on daily outpatient visits for AS at lag 0-7 day; A nature cubic spline with 3 dfs was used to control environmental factors, 7 dfs per year was used to control the seasonal patterns and long term trends.


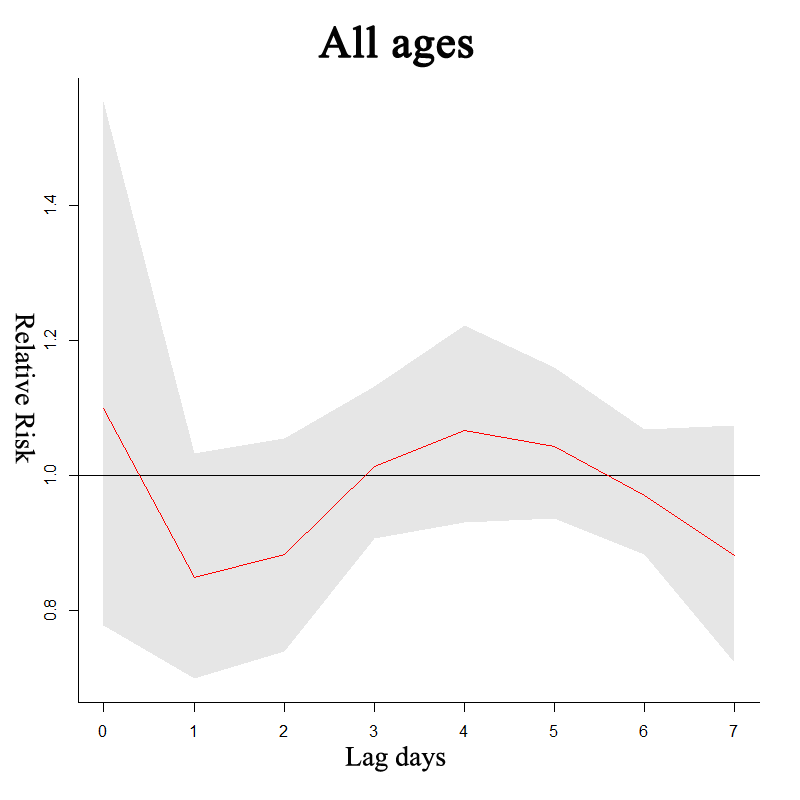


**Fig. S7** Effects for hot weather (99st percentiles of temperature) on daily outpatient visits for AS at lag 0-7 day; A nature cubic spline with 3 dfs was used to control environmental factors, 9 dfs per year was used to control the seasonal patterns and long term trends.


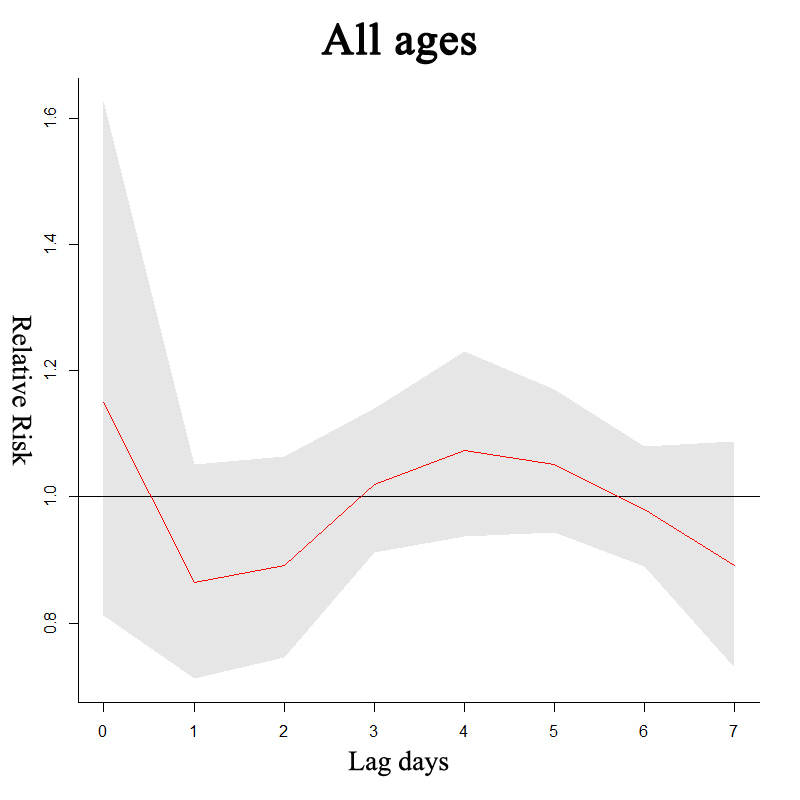


**Fig. S8** Effects for hot weather (99st percentiles of temperature) on daily outpatient visits for AS at lag 0-7 day; A nature cubic spline with 4 dfs was used to control environmental factors, 8 dfs per year was used to control the seasonal patterns and long term trends.


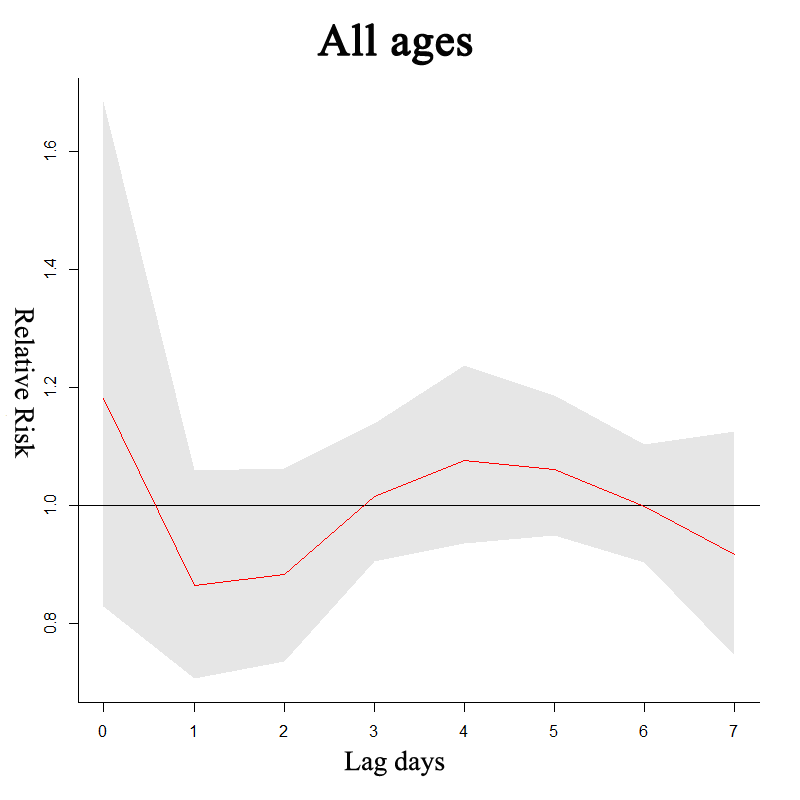


**Fig. S9** Effects for hot weather (99st percentiles of temperature) on daily outpatient visits for AS at lag 0-7 day; A nature cubic spline with 5 dfs was used to control environmental factors, 8 dfs per year was used to control the seasonal patterns and long term trends.


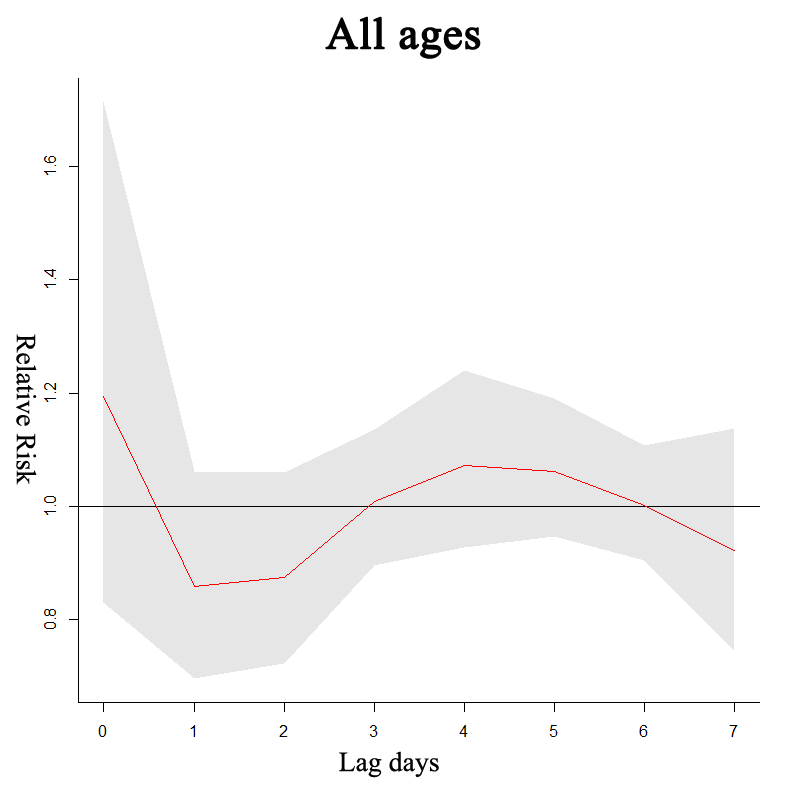


**Fig. S10** Effects for dry weather (1st percentiles of relative humidity) on daily outpatient visits for AS at lag 0-7 day; A nature cubic spline with 3 dfs was used to control environmental factors, 7 dfs per year was used to control the seasonal patterns and long term trends.


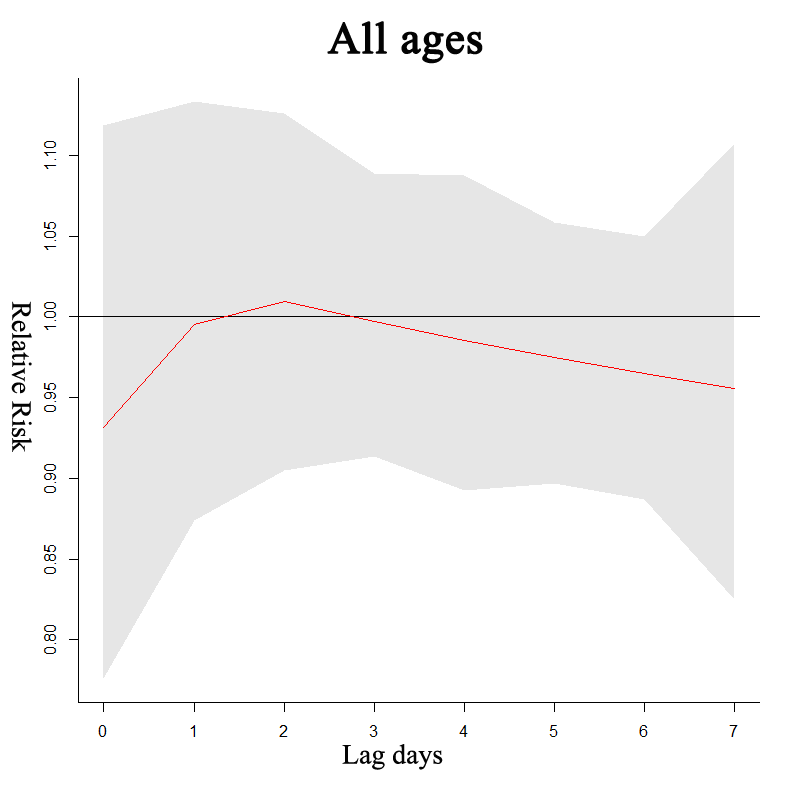


**Fig. S11** Effects for dry weather (1st percentiles of relative humidity) on daily outpatient visits for AS at lag 0-7 day; A nature cubic spline with 3 dfs was used to control environmental factors, 9 dfs per year was used to control the seasonal patterns and long term trends.


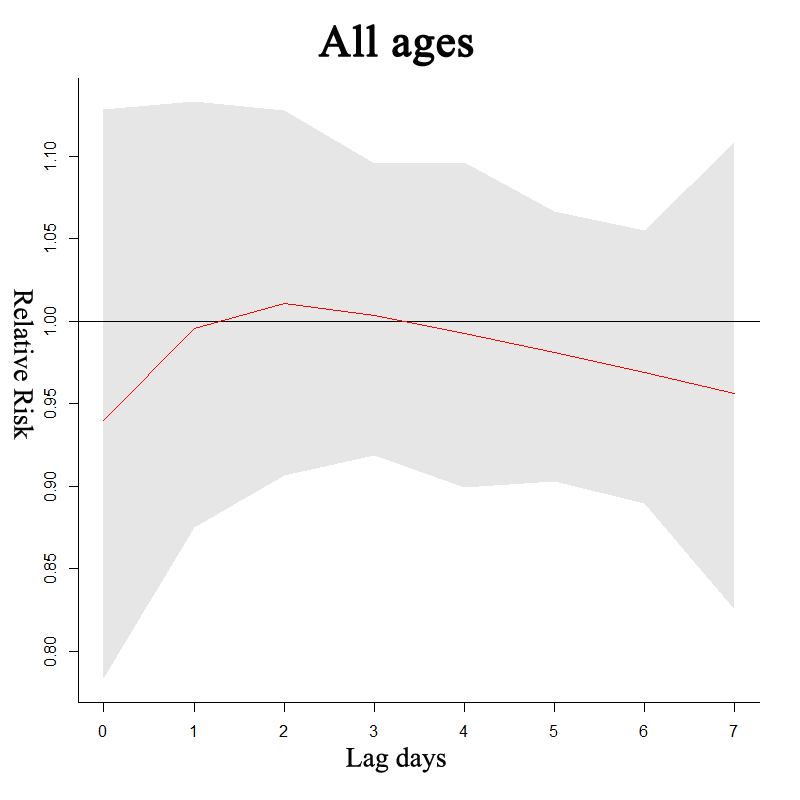


**Fig. S12** Effects for dry weather (1st percentiles of relative humidity) on daily outpatient visits for AS at lag 0-7 day; A nature cubic spline with 4 dfs was used to control environmental factors, 8 dfs per year was used to control the seasonal patterns and long term trends.


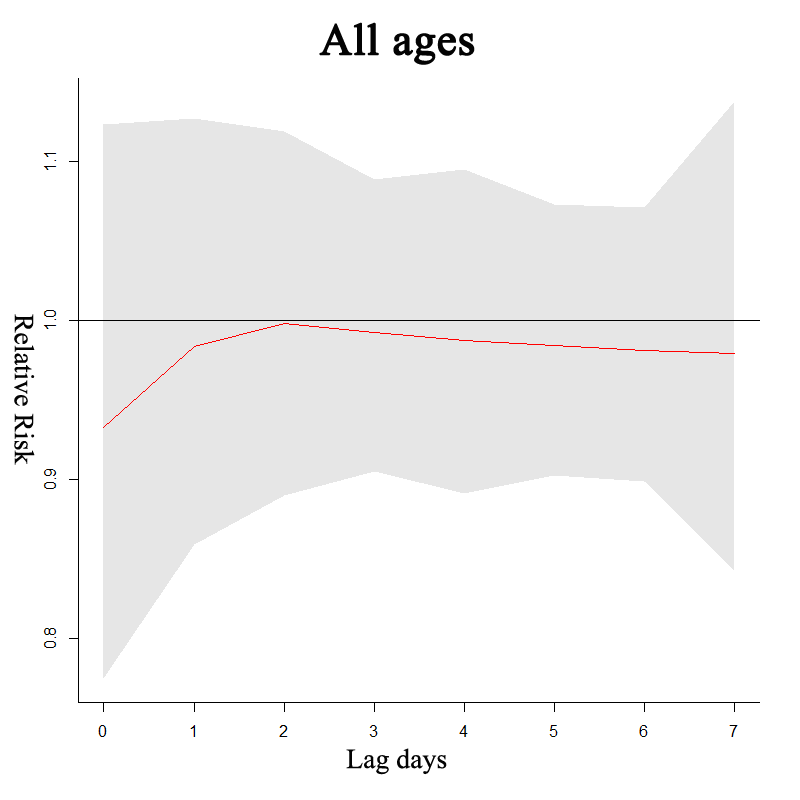


**Fig. S13** Effects for dry weather (1st percentiles of relative humidity) on daily outpatient visits for AS at lag 0-7 day; A nature cubic spline with 5 dfs was used to control environmental factors, 8 dfs per year was used to control the seasonal patterns and long term trends.


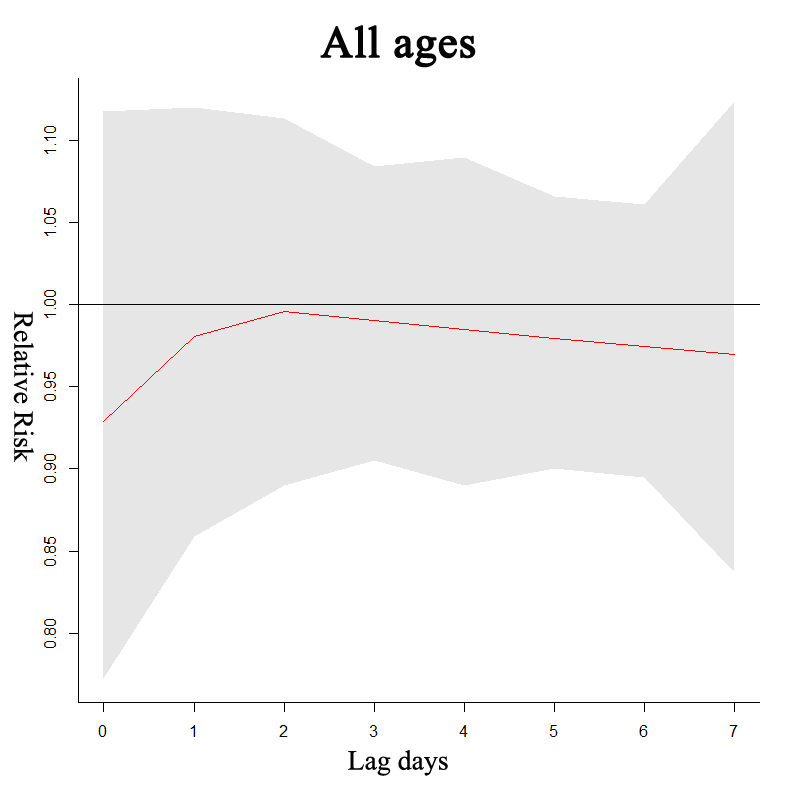


**Fig. S14** Effects for damp weather (99th percentiles of relative humidity) on daily outpatient visits for AS at lag 0-7 day; A nature cubic spline with 3 dfs was used to control environmental factors, 7 dfs per year was used to control the seasonal patterns and long term trends.


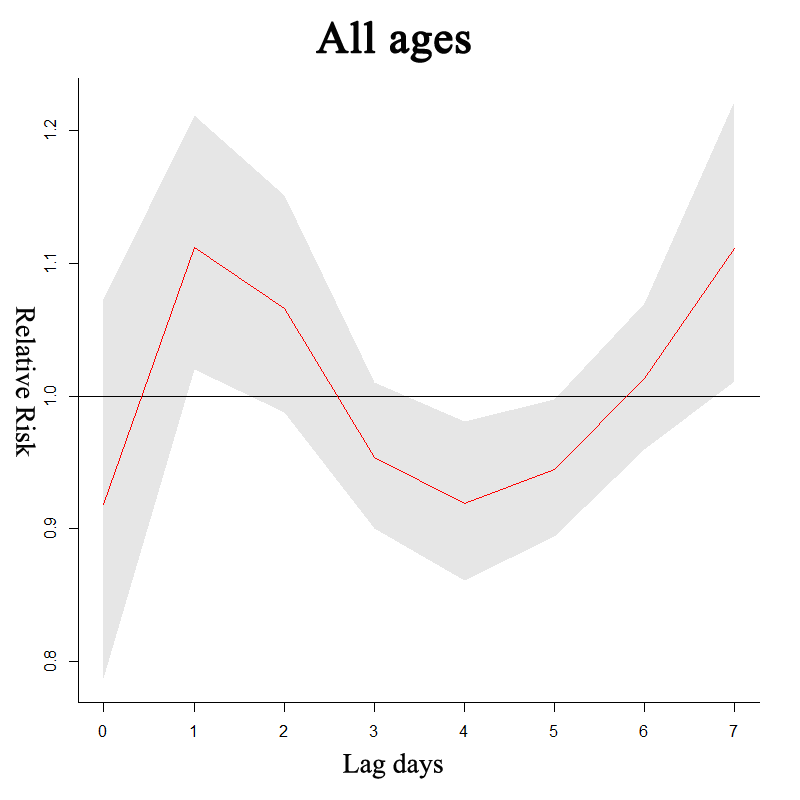


**Fig. S15** Effects for damp weather (99th percentiles of relative humidity) on daily outpatient visits for AS at lag 0-7 day; A nature cubic spline with 3 dfs was used to control environmental factors, 9 dfs per year was used to control the seasonal patterns and long term trends.


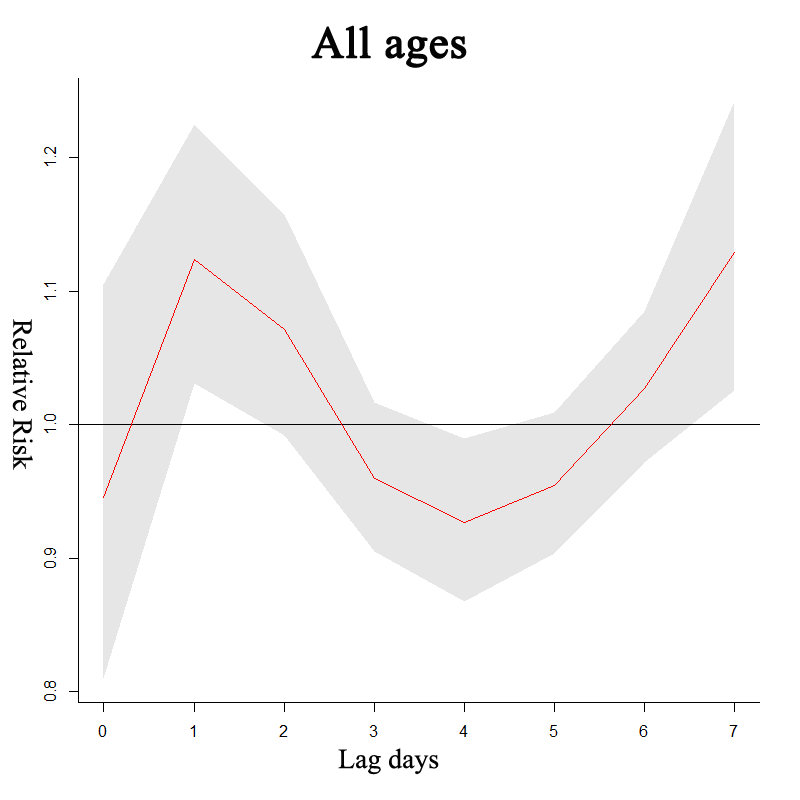


**Fig. S16** Effects for damp weather (99th percentiles of relative humidity) on daily outpatient visits for AS at lag 0-7 day; A nature cubic spline with 4 dfs was used to control environmental factors, 8 dfs per year was used to control the seasonal patterns and long term trends.


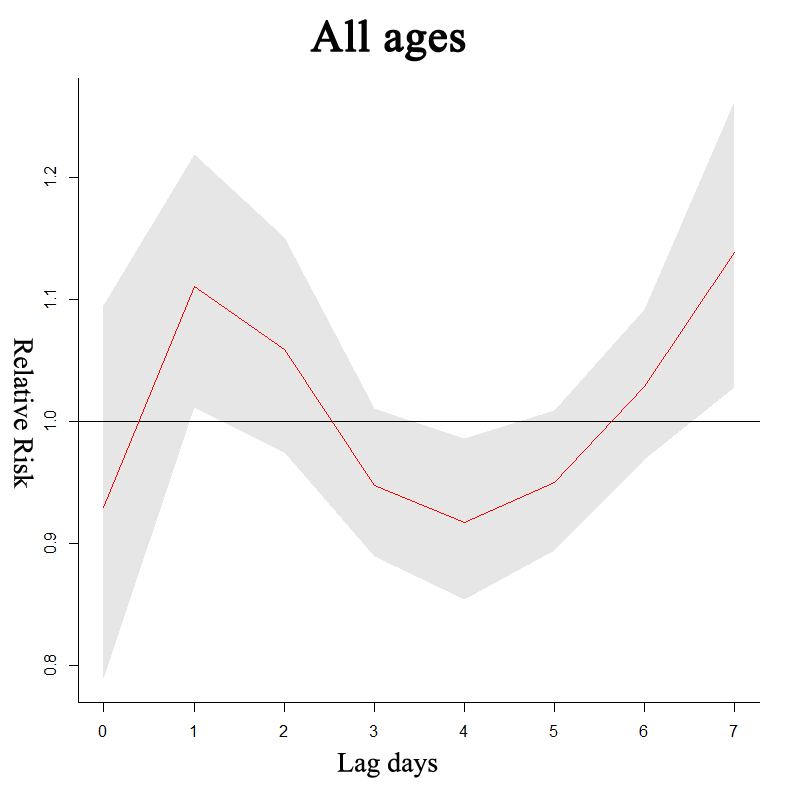


**Fig. S17** Effects for damp weather (99th percentiles of relative humidity) on daily outpatient visits for AS at lag 0-7 day; A nature cubic spline with 5 dfs was used to control environmental factors, 8 dfs per year was used to control the seasonal patterns and long term trends.


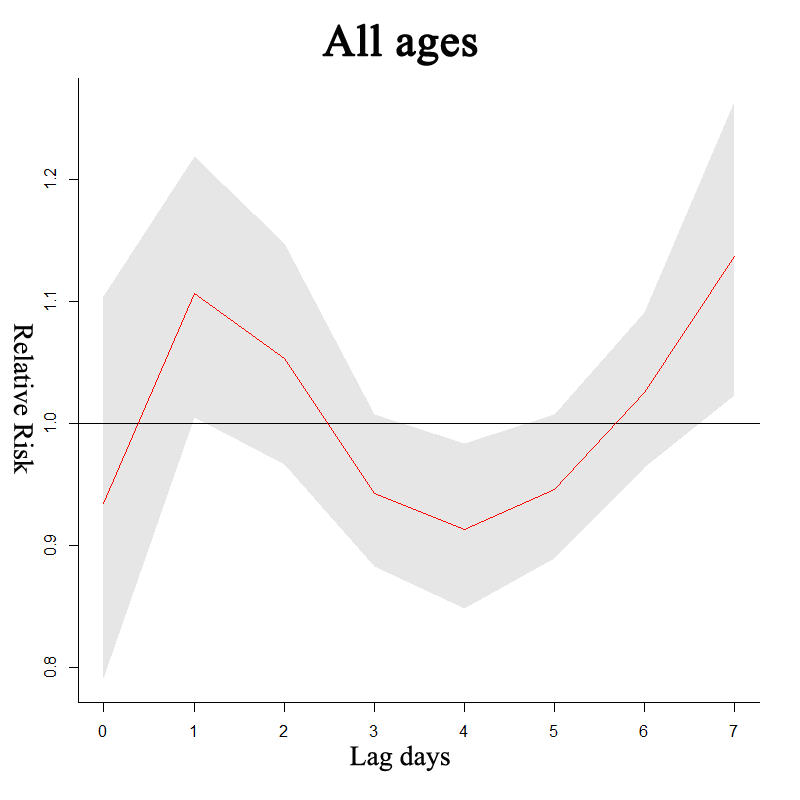

Supplement: Supplementary file 1 — Additional file 1: Fig. S1. Data selection process. Fig. S2. Effects for cold weather (1st percentiles of temperature) on daily outpatient visits for AS at lag 0–7 day; A nature cubic spline with 3 dfs was used to control environmental factors, 7 dfs per year was used to control the seasonal patterns and long term trends. Fig. S3. Effects for cold weather (1st percentiles of temperature) on daily outpatient visits for AS at lag 0–7 day; A nature cubic spline with 3 dfs was used to control environmental factors, 9 dfs per year was used to control the seasonal patterns and long term trends. Fig. S4. Effects for cold weather (1st percentiles of temperature) on daily outpatient visits for AS at lag 0–7 day; A nature cubic spline with 4 dfs was used to control environmental factors, 8 dfs per year was used to control the seasonal patterns and long term trends. Fig. S5. Effects for cold weather (1st percentiles of temperature) on daily outpatient visits for AS at lag 0–7 day; A nature cubic spline with 5 dfs was used to control environmental factors, 8 dfs per year was used to control the seasonal patterns and long term trends. Fig. S6. Effects for hot weather (99st percentiles of temperature) on daily outpatient visits for AS at lag 0–7 day; A nature cubic spline with 3 dfs was used to control environmental factors, 7 dfs per year was used to control the seasonal patterns and long term trends. Fig. S7. Effects for hot weather (99st percentiles of temperature) on daily outpatient visits for AS at lag 0–7 day; A nature cubic spline with 3 dfs was used to control environmental factors, 9 dfs per year was used to control the seasonal patterns and long term trends. Fig. S8. Effects for hot weather (99st percentiles of temperature) on daily outpatient visits for AS at lag 0–7 day; A nature cubic spline with 4 dfs was used to control environmental factors, 8 dfs per year was used to control the seasonal patterns and long term trends. Fig. S9. Effects for hot weather (99st perce [file 12891_2021_4523_MOESM1_ESM.doc]
