# Supplementary material for: Exposure-lag-response associations between weather conditions and ankylosing spondylitis: a time series study
Source: BMC Musculoskelet Disord. 2021 Jul 26;22:641. doi: 10.1186/s12891-021-04523-y (PMC8314534; doi:10.1186/s12891-021-04523-y)
Supplement: Supplementary file 2 — Additional file 2: Table S1. Relative risk (RR) and 95% confidence intervals (95% CI) for low and high temperatures associated with daily AS outpatient over different lag days in Hefei, China, from January 2014 to December 2019. Table S2. Relative risk (RR) and 95% confidence intervals (95% CI) for low and high relative humidity associated with daily AS outpatient visits over different lag days in Hefei, China, from January 2014 to December 2019. [file 12891_2021_4523_MOESM2_ESM.docx]

**Table S1.** Relative risk (RR) and 95% confidence intervals (95% CI) for low and high temperatures associated with daily AS outpatient over different lag days in Hefei, China, from January 2014 to December 2019

| Group | Low temperature (-1 °C) | | | | | | | |
| --- | --- | --- | --- | --- | --- | --- | --- | --- |
|  | Lag 0 days | Lag 1 days | Lag 2 days | Lag 3 days | Lag 4 days | Lag 5 days | Lag 6 days | Lag 7 days |
| All | 0.850  (0.578-1.249) | 1.032  (0.858-1.243) | 1.054  (0.889-1.249) | 1.005  (0.906-1.114) | 0.987  (0.869-1.121) | 0.998  (0.901-1.104) | 1.027  (0.939-1.124) | 1.068  (0.887-1.286) |
| Male | 0.755  (0.480-1.188) | 1.037  (0.834-1.290) | 1.125  (0.921-1.375) | 1.089  (0.966-1.228) | 1.064  (0.917-1.234) | 1.052  (0.935-1.185) | 1.050  (0.945-1.167) | 1.053  (0.847-1.308) |
| Female | 1.013  (0.521-1.968) | 1.112  (0.810-1.527) | 0.989  (0.744-1.316) | 0.851  (0.709-1.020) | 0.821  (0.654-1.029) | 0.871  (0.728-1.042) | 0.985  (0.842-1.153) | 1.151  (0.832-1.592) |
| ≥65 years | 0.444  (0.063-3.150) | 0.907  (0.360-2.284) | 0.962  (0.419-2.209) | 0.811  (0.484-1.359) | 0.802  (0.421-1.526) | 0.913  (0.548-1.522) | 1.143  (0.716-1.824) | 1.499  (0.572-3.926) |
| <65 years | 0.814  (0.552-1.200) | 1.093  (0.908-1.316) | 1.119  (0.945-1.325) | 1.029  (0.927-1.142) | 0.990  (0.870-1.128) | 0.996  (0.898-1.104) | 1.030  (0.940-1.128) | 1.081  (0.897-1.304) |
|  | High temperature (32.9 °C) | | | | | | | |
| All | 1.063  (0.754-1.499) | 0.894  (0.734-1.090) | 0.929  (0.775-1.113) | 1.028  (0.919-1.150) | 1.063  (0.928-1.218) | 1.038  (0.932-1.156) | 0.974  (0.883-1.074) | 0.897  (0.734-1.095) |
| Male | 1.323  (0.889-1.968) | 0.908  (0.723-1.141) | 0.898  (0.729-1.106) | 1.013  (0.890-1.153) | 1.055  (0.901-1.234) | 1.021  (0.901-1.156) | 0.941  (0.841-1.053) | 0.846  (0.672-1.067) |
| Female | 0.649  (0.349-1.206) | 0.796  (0.558-1.135) | 0.938  (0.679-1.296) | 1.043  (0.852-1.276) | 1.092  (0.854-1.396) | 1.092  (0.898-1.327) | 1.059  (0.887-1.265) | 1.013  (0.705-1.455) |
| ≥65 years | 2.678  (0.512-14.002) | 1.079  (0.410-2.842) | 0.687  (0.289-1.635) | 0.643  (0.379-1.090) | 0.768  (0.396-1.487) | 1.096  (0.646-1.858) | **1.761**  **(1.130-2.744)** | **3.004**  **(1.201-7.510)** |
| <65 years | 1.058  (0.747-1.499) | 0.847  (0.694-1.033) | 0.897  (0.748-1.074) | 1.031  (0.920-1.155) | 1.079  (0.939-1.238) | 1.041  (0.933-1.162) | 0.953  (0.863-1.053) | 0.849  (0.692-1.041) |

Note. A nature cubic spline with 3 df was used to control relative humidity, wind speed, atmospheric pressure, and rainfall; Statistically significant results (P < 0.05) are shown in bold font. The reference values are the mean value of temperature (MTEM: 16.9 °C), and the effects of low and high temperature were estimated by calculating the risk of an AS outpatient visit at the 1st and 99th percentiles of temperature relative to the MTEM, respectively.

**Table S2.** Relative risk (RR) and 95% confidence intervals (95% CI) for low and high relative humidity associated with daily AS outpatient visits over different lag days in Hefei, China, from January 2014 to December 2019

| Group | Low relative humidity (42%) | | | | | | | |
| --- | --- | --- | --- | --- | --- | --- | --- | --- |
|  | Lag 0 days | Lag 1 days | Lag 2 days | Lag 3 days | Lag 4 days | Lag 5 days | Lag 6 days | Lag 7 days |
| All | 0.938  (0.781-1.127) | 0.992  (0.872-1.129) | 1.006  (0.902-1.122) | 0.998  (0.915-1.090) | 0.989  (0.895-1.091) | 0.978  (0.900-1.063) | 0.968  (0.889-1.053) | 0.957  (0.826-1.109) |
| Male | 0.978  (0.791-1.209) | 0.937  (0.801-1.095) | 0.944  (0.827-1.077) | 0.967  (0.871-1.073) | 0.977  (0.869-1.099) | 0.976  (0.884-1.076) | 0.966  (0.874-1.068) | 0.953  (0.801-1.133) |
| Female | 0.859  (0.620-1.190) | 1.102  (0.893-1.359) | 1.134  (0.951-1.353) | 1.059  (0.914-1.226) | 1.009  (0.854-1.193) | 0.983  (0.855-1.131) | 0.972  (0.842-1.123) | 0.968  (0.753-1.245) |
| ≥65 years | 0.337  (0.095-1.196) | 1.065  (0.564-2.010) | 1.396  (0.815-2.393) | 1.187  (0.765-1.841) | 1.025  (0.620-1.694) | 0.916  (0.602-1.395) | 0.838  (0.540-1.302) | 0.776  (0.357-1.685) |
| <65 years | 0.957  (0.796-1.151) | 0.989  (0.867-1.127) | 0.998  (0.893-1.114) | 0.993  (0.909-1.086) | 0.987  (0.893-1.091) | 0.979  (0.901-1.065) | 0.971  (0.891-1.058) | 0.962  (0.829-1.117) |
|  | High relative humidity (98%) | | | | | | | |
| All | 0.925  (0.792-1.081) | **1.113**  **(1.021-1.213)** | 1.066  (0.987-1.151) | 0.954  (0.900-1.011) | **0.920**  **(0.862-0.983)** | 0.947  (0.896-1.000) | 1.017  (0.963-1.074) | **1.115**  **(1.014-1.227)** |
| Male | 0.913  (0.763-1.092) | **1.141**  **(1.033-1.260)** | 1.080  (0.988-1.181) | 0.945  (0.883-1.011) | **0.909**  **(0.842-0.981)** | 0.948  (0.889-1.010) | 1.044  (0.980-1.111) | **1.181**  **(1.058-1.317)** |
| Female | 0.948  (0.718-1.251) | 1.055  (0.905-1.229) | 1.039  (0.906-1.191) | 0.979  (0.884-1.085) | 0.949  (0.846-1.065) | 0.943  (0.856-1.039) | 0.953  (0.864-1.052) | 0.971  (0.818-1.153) |
| ≥65 years | 1.032  (0.474-2.245) | 0.838  (0.537-1.308) | 0.755  (0.508-1.121) | 0.739  (0.543-1.007) | 0.761  (0.538-1.077) | 0.813  (0.608-1.087) | 0.889  (0.672-1.177) | 0.985  (0.608-1.595) |
| <65 years | 0.922  (0.788-1.079) | **1.123**  **(1.029-1.225)** | 1.077  (0.996-1.164) | 0.961  (0.906-1.019) | **0.925**  **(0.865-0.988)** | 0.950  (0.899-1.005) | 1.020  (0.965-1.078) | **1.120**  **(1.017-1.233)** |

Note. A nature cubic spline with 3 df was used to control temperature, wind speed, atmospheric pressure, and rainfall. Statistically significant results (P < 0.05) are shown using bold font. The reference values are the mean value of relative humidity (MRHU: 75.02%). The effects of low and high relative humidity were estimated by calculating the risk of AS outpatient at the 1st and 99th percentiles of relative humidity relative to the MRHU, respectively.
